# Study of banana preservation extension by UVC radiation in precise monitoring LED irradiation cavity

**DOI:** 10.1038/s41598-022-25716-y

**Published:** 2022-12-09

**Authors:** Thi-Thu-Ngoc Le, Chung-Ta Liao, Shih-Kang Lin, Chi-Shou Wu, Quang-Khoi Nguyen, Tsung-Hsun Yang, Yeh-Wei Yu, Ching-Cherng Sun

**Affiliations:** 1 Department of Optics and Photonics, National Central University, Chung-Li, 32054 Taiwan; 2 Crop Environment Section, Taichung District Agricultural Research and Extension Station, COA, Changhua County, 51544 Taiwan; 3 Department of Electrophysics, National Yang-Ming Chiao Tung, Hsin-Chu, 30010 Taiwan

**Keywords:** Lasers, LEDs and light sources, Optical techniques

## Abstract

Ultraviolet C (UVC) radiation has been considered a possible option to alleviate the seriousness of black spots on bananas during preservation which help increase economic efficiency. In this study, using 275 nm UVC light-emitting diodes (LEDs), a preliminary cavity with dimensions of 30 × 30 × 30 cm was designed and fabricated to aid in reducing black spots on bananas with the aim of application in the factory conveyor belts. The UVC irradiance distribution was thoroughly monitored for many sections at different box heights in both simulation and measurement, with a dominant range of 6–9 W/m^2^ in the middle. Afterward, trials were conducted in vitro and in vivo at different selected UVC doses. The results in vitro revealed that a dose of over 0.36 kJ/m^2^ has an excellent effect on inhibiting the colonial germination of fungal *Colletotrichum musae*, a common species of fungi causing black spot disease on bananas. In vivo conditions, with a short exposure time of around 5 s, the black spots on UVC-irradiated banana peel significantly reduced with minimal sensory damage compared to a control banana via observation after seven days from treatment. Finally, the optimal UVC dose is proposed from 0.030 to 0.045 kJ/m^2^ for the one-time treatment when considering the upper surface of the banana. With flexibility advantage and short exposure time, the fabricated cavity (box) promises to bring a lot of application potential to aid banana preservation in factories and households.

## Introduction

Treatments based on UVC radiation (200–280 nm wavelength) could be considered a potential option for postharvest preservation instead of chemical controls that cause hazards to health and the environment^1–10^. Indeed, UVC irradiation has been demonstrated to inactivate bacteria and fungi on fruits^11–16^. Further, UVC irradiation can enhance the resistance mechanism of fruits and improve antioxidant levels^17,18^. Many people desire a germicidal product to aid fruit preservation with high effectiveness, flexibility, and convenience that could be applied in factories or households. The UVC LED is a potential candidate for fabricating sterilization products more flexibly due to its compact size compared with traditional lamps. Besides, UVC LEDs have environmental benefits, lower energy costs, long life spans, and low power consumption^19,20^. Therefore, the fabrication of sterilization products using UVC LEDs to aid fruit preservation has more advantages that have not been widely studied.

Among many kinds of fruits, the banana is one of the most popular desserts worldwide that is highly susceptible to perishable during transportation and storage^21,22^. Anthracnose disease, caused by the fungus *Colletotrichum musae* (*C. musae*), is the most common postharvest disease, with the symptoms as black or brown spots of different sizes on their peel^23,24^. For germicidal to be effective, applying a suitable UVC dose is crucial during the treatment process. Otherwise, the over UVC dose may damage the sensory properties of banana peels according to unexpected aspects, especially the peel color may change to brown. The UVC dose (*D*_*UVC*_) is calculated via the formula as

1 $${{D}}_{{UVC}}= {{E}} \, \cdot \, {{t}}$$

where the unit of irradiance (*E*) is W/m^2^ and the exposure time (*t*) is second (s), so the unit of dose is W s/m^2^ or J/m^2^. We can control the irradiance or exposure time to achieve a desirable dose. Therefore, monitoring irradiance distribution is important in designing and fabricating a particular product. To the best of our knowledge, there are several reports of interest in the UVC light effects in expanding the shelf life of banana fruit after harvest^25–27^ but fabricating a specific product by using 275 nm UVC LEDs with thorough monitoring of irradiance distribution that uses for postharvest fruits preservation, especially for banana, have not been presented yet.

In this work, we built up a preliminary UVC LED box with dimensions of 30 $\times$ 30 $\times$ 30 cm, which has high potential application in aid of banana preservation not only in industrial conveyor belts of factories but also in households or retailers. To predict and control the optical behavior and fabricate a sterilization box as desirable, we need to design the box and then fabricate it. We started by collecting UVC LEDs with wavelengths of around 275 nm and then built up the optical modeling, which is a necessary step before lighting design. After we achieved the precise model that can apply to predict the optical behavior effectively, we used this model to design a box so that the UVC irradiation focuses in the middle region of the box and has high irradiance uniformity. Afterward, we attempt to fabricate an actual box similar to the design by arranging UVC LED to the box covered by Aluminum plates. The UVC irradiance was monitored both in simulation and in experiment carefully for many cross-sections at different heights of the box, then the comparison between them was thoroughly conducted. The UVC box effect is studied under in vitro and in vivo conditions by treatment at different selected UVC doses. In vitro, the UVC box effect is studied on *C. musae*, and the objective is to observe and confirm the inhibited effects on the fungi species. In vivo, bananas are treated by locating them one by one in the box middle with different exposure times and via daily evaluation to figure out the optimal UVC dose. The study will be respectively detailed in the following sections.

## Optical modeling of UVC LED

**Figure 1:**
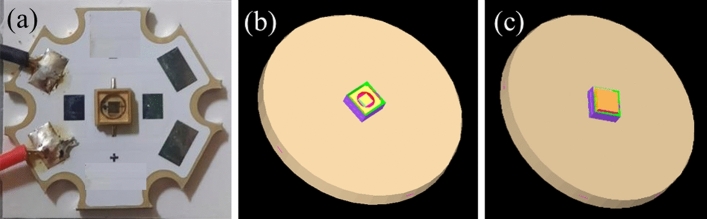
(**a**) UVC LED sample used for the experiment, (**b**) optical model for UVC LED without quartz window, and (**c**) with a quartz window.

It is essential to own the light source model accurately before proceeding with a design to predict optical performances. It is started by collecting 275 nm UVC LEDs with the geometry shown in Fig. 1a, where a transparent quartz window covers the LED cavity. Its radiant flux was measured around 46 mW with an applied current of 0.35 A. Here the optical model of this light source was well done, in which all verification was conducted at the mid-field distances from the light source^28–30^.

We initially created a model by setting the most crucial optical parameters, including the geometry of the components, the material properties, and the weighting factors of the emitting area^28,31^. The geometry of the light source model was built as shown in Fig. 1b without a quartz window and (c) with a quartz window. Following this, we simulated the light patterns by applying the Monte-Carlo ray-tracing methodology in the ASAP program^32^. In this case, we did the light source model by comparing the 2-dimension (2-D) light pattern in several mid-field distances that were not larger than ten times the largest lateral dimension of the LED light source^29^. Because UVC light is invisible light, a fluorescent film was applied to absorb UVC light and re-emit visible light then a mono-CMOS sensor was used to capture the light pattern. According to the result, the simulated 2-D light patterns and corresponding measurements were virtually similar at several mid-field distances. The normalized cross-section (NCC) between two light patterns always reached more than 99.5%, meaning this light source model is precise enough to apply for lighting design^30^.

### Policy and plant use guidelines

The authors confirm that the bananas used in this present study was in accordance to international, national and/or institutional guidelines.

## The UVC LED box and irradiance monitoring

### The UVC box and irradiance uniformity analysis

**Figure 2:**
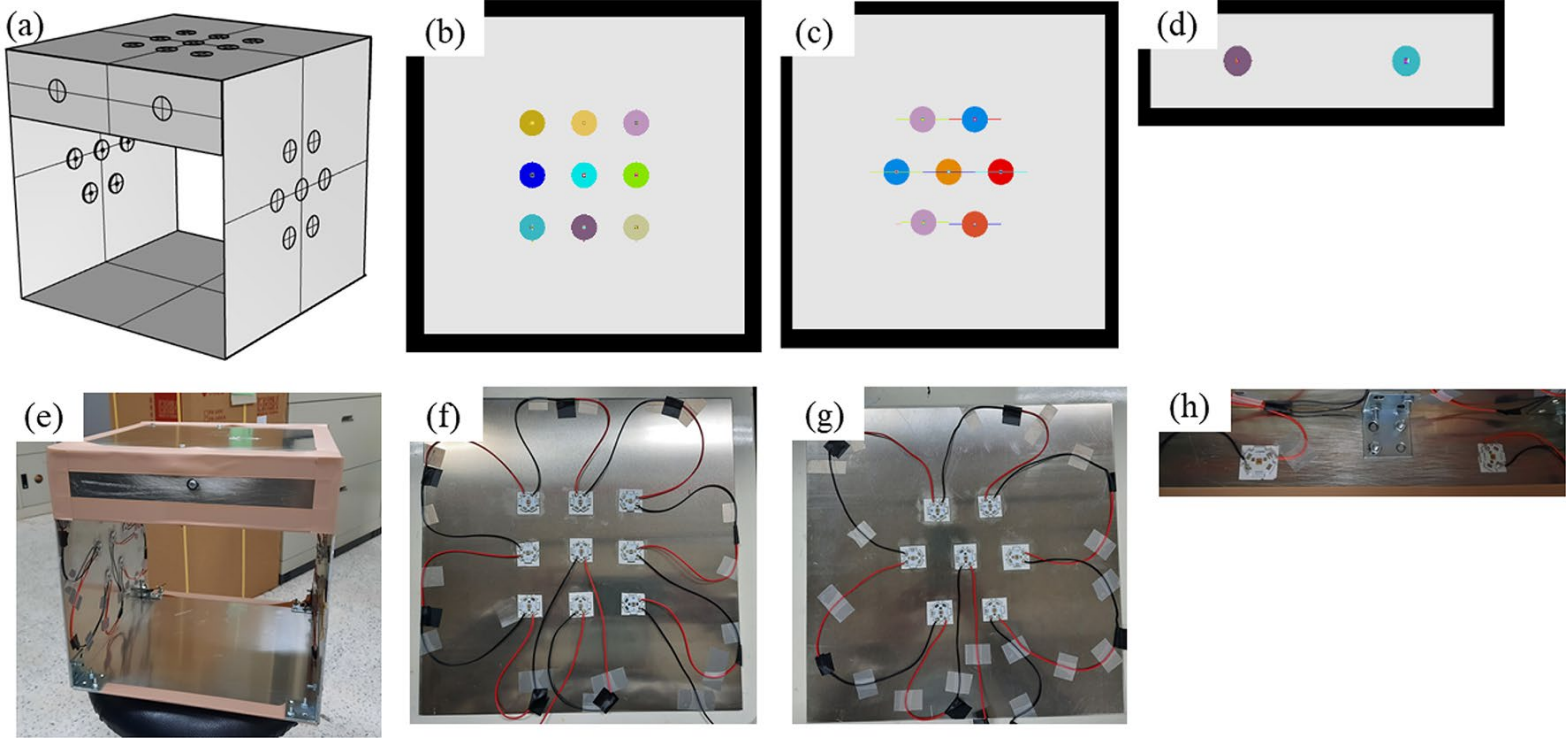
Arrangement of UVC LED for (**a**) whole box, (**b**) top plate, (**c**) side plate, (**d**) sidebar in simulation; arrangement of UVC LED for (**e**) whole box, (**f**) top plate, (**g**) side plate, (**h**) sidebar in experiment.

The preliminary UVC box was designed so that the irradiation could focus on the middle region, as shown in Fig. 2a–d. Because the heat problem of LEDs is vital^33,34^, to ensure irradiance must reach thermal equilibrium, the Aluminum material is selected to make the box covers to aid the heat dispersion. Besides, the LEDs are kept from heating up during the experiments. The UVC LED was mainly arranged on three plates with nine LEDs on the top plate and seven LEDs on each two side plates. We kept the two remaining sides of the box with empty covers so that fruit flow could circulate through the middle of the box for application in industrial factory conveyor belts. Also, the box could be applied in households if it is additionally covered to protect users from UVC damage. To enhance the irradiation for two sides with empty covers, we designed two sidebars and arranged two LEDs on each side. In this case, the bottom was not filled with LEDs to hold the banana. During the design procedure, we analyzed the irradiance uniformity of the middle section to control the distance between LEDs. The middle section with dimensions of around 21 $\times$ 21 cm is the target section facing the box top plate, which can receive light from the top-down and side plates.

**Figure 3:**
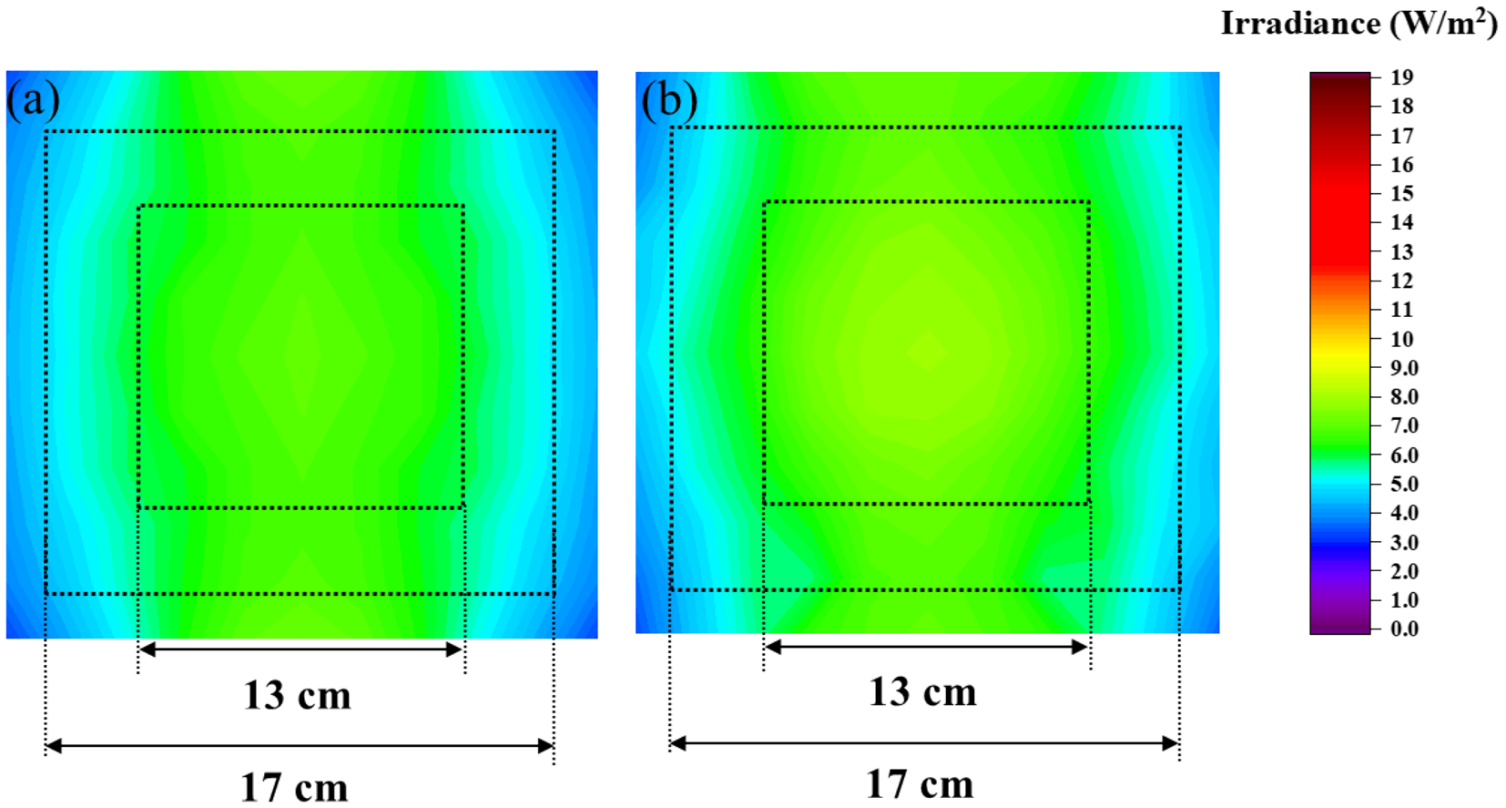
The light pattern of the middle section in (**a**) simulation and (**b**) experiment.

We calculate the irradiance uniformity $U$ of the target section by applying the formula as.

2 $$U = \frac{{E_{Min} }}{{E_{Ave} }}\times100\%,$$

where ${E}_{Min}$ (W/m^2^) is the minimum irradiance and ${E}_{Ave}$ (W/m^2^) is the average irradiance on the target section^35^. For the simulated irradiance section with dimensions 21 $\times$ 21 cm in Fig. 3a, the uniformity was calculated for squares with dimensions 13 $\times$ 13 cm and 17 $\times$ 17 cm as 87.1% and 77.0%, corresponding. As a result, it was predicted that the irradiance distribution with high uniformity in the middle section. After, we tried to fabricate the reality box similar to the simulation, as shown in Fig. 2e–h. Using a detector connected with a power meter, we measured the irradiance on the target section by facing the detector to the box top and moving to many positions; the irradiance pattern as described in Fig. 3b, the UVC uniformity was also calculated for the measured irradiance square with dimensions 13 $\times$ 13 cm and 17 $\times$ 17 cm as 83.3% and 73.2%, corresponding.

### 3–2 Irradiance distribution

**Figure 4:**
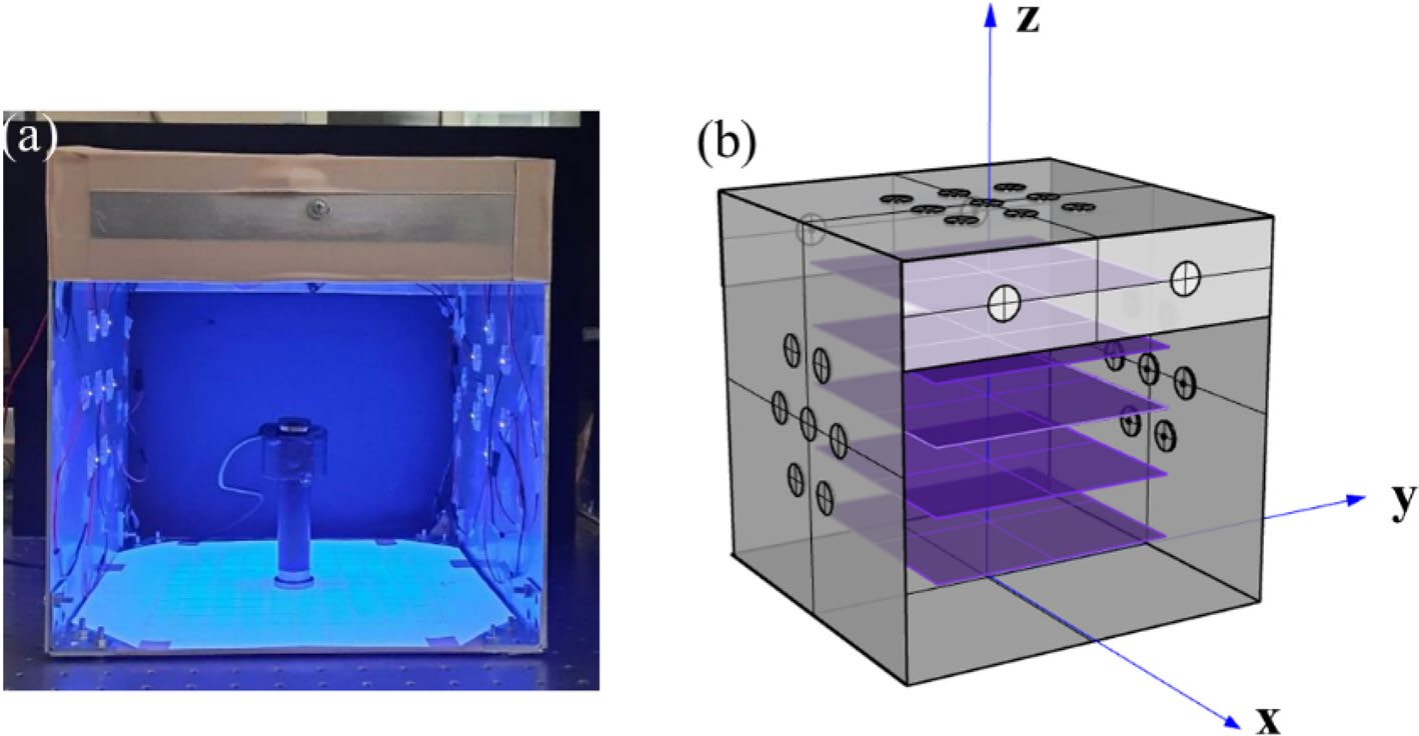
(**a**) The photo of the irradiance measurement, and (**b**) description of several measured sections.

**Figure 5:**
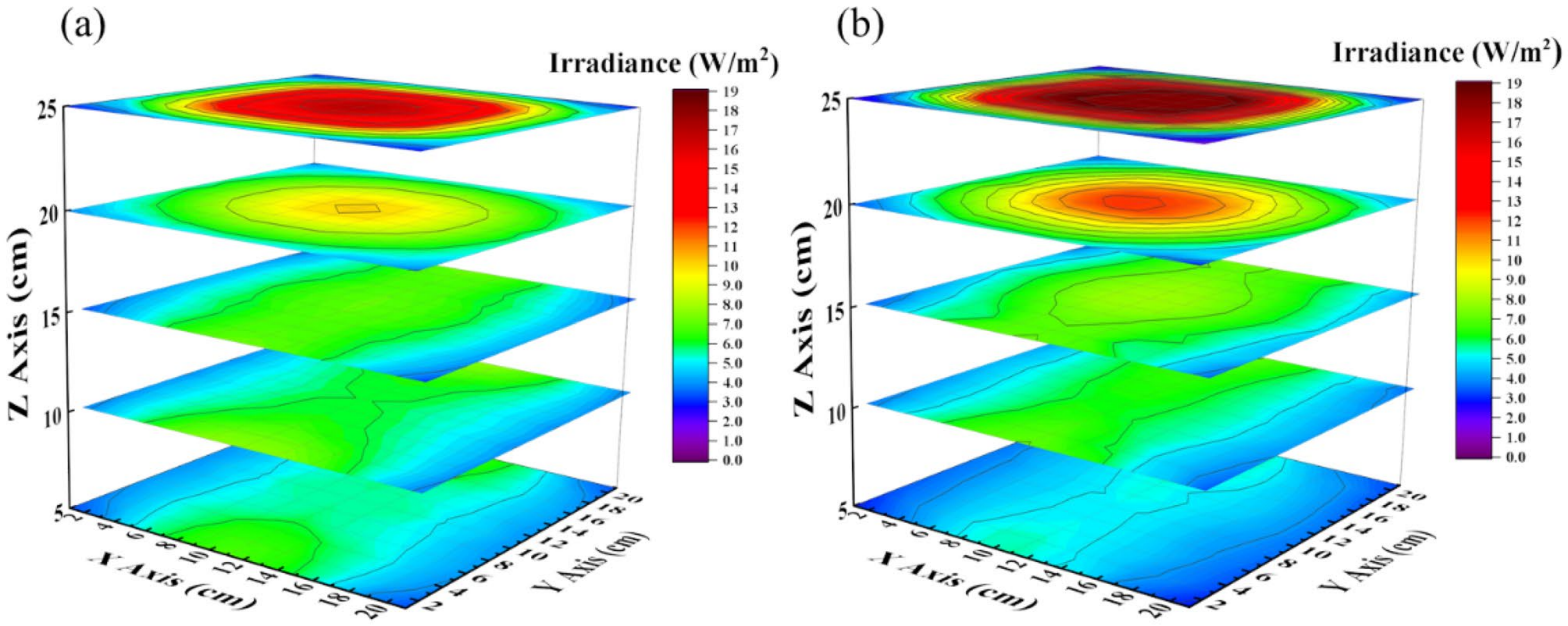
The light pattern of the section at different positions in (**a**) simulation, and (**b**) experiment; the section receive light from the top surface illuminating downwards and from the sides.

For more analysis of the irradiance distribution in the box, we continually monitored its distribution on several sections at different box heights both in simulation and in the experiment. In the experiment, we adjusted the height of the detector and moved it to many positions to measure irradiance, where the detector faces the box top in Fig. 4a, merging all locations of the detector at the same height forms each section which receives the light from the top of the box downward and side plates, as depicted in Fig. 4b. Figure 5a,b shows the irradiance distribution of sections at different heights in simulation and measurement. The results indicated that irradiance gradually decrease from the top to the bottom and more uniformity in the middle section. In general, the irradiance distribution in both simulation and measurement are virtually similar to each other.

**Figure 6:**
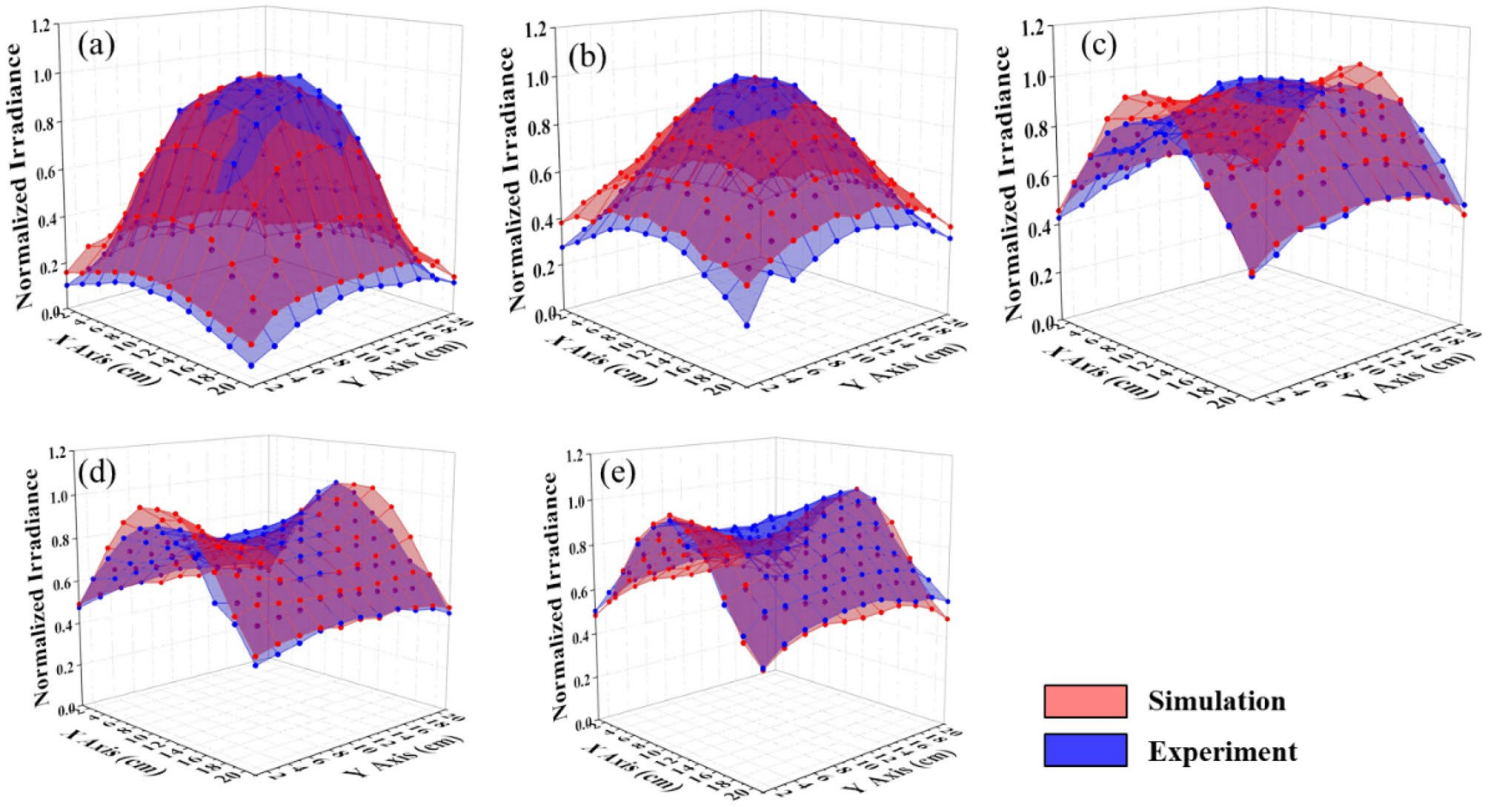
Comparison of the irradiance distribution of sections between simulation and experiment in normalization at different heights (**a**) 25, (**b**) 20, (**c**) 15, (**d**) 10, and (**e**) 5 cm with NCC as 99.7, 99.2, 97.0, 95.1, and 95.6%, respectively.

**Figure 7:**
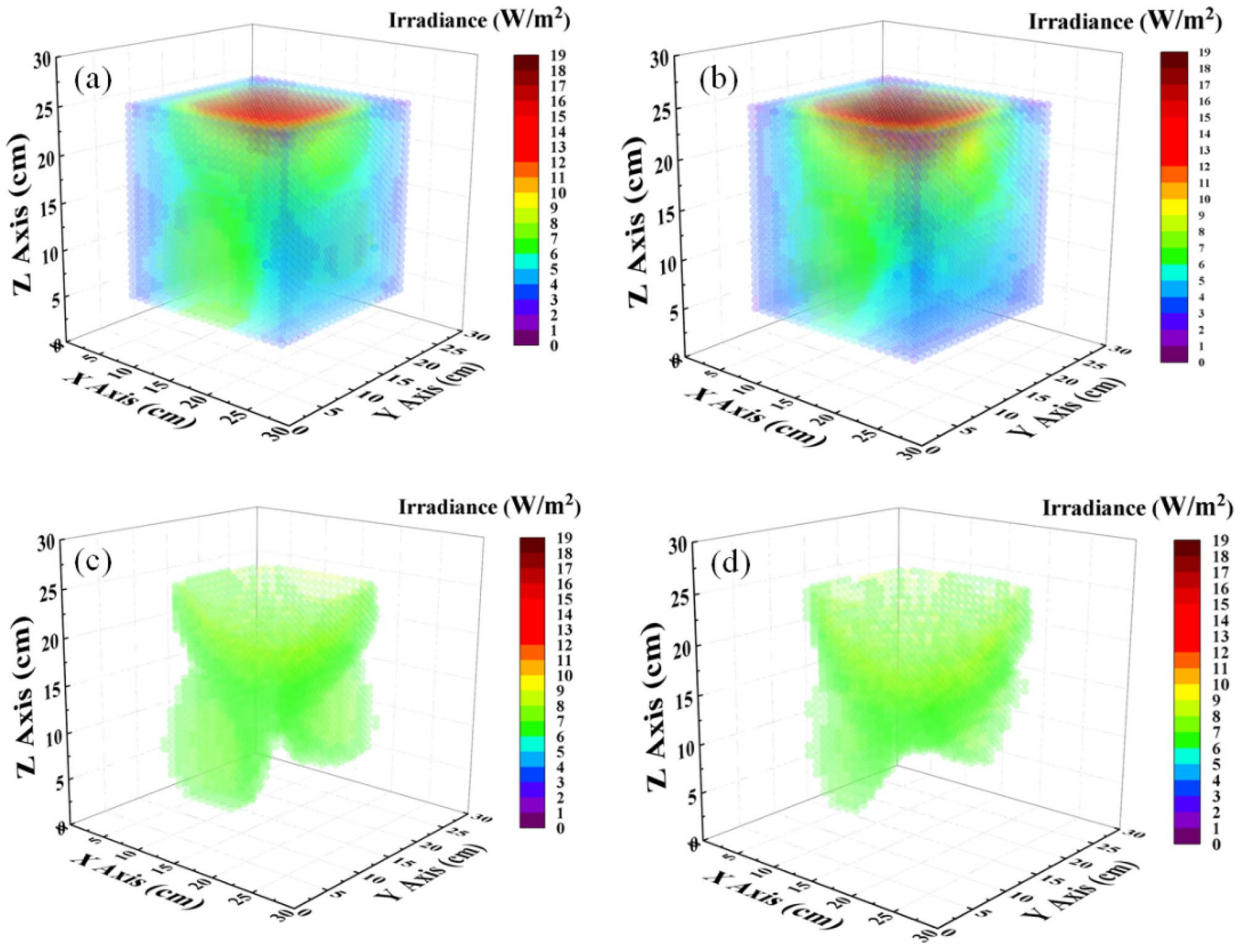
Irradiance distribution of multi-section inside the box in (**a**) simulation and (**b**) experiment; the filtered irradiance distribution with a range from 6 to 9 W/m^2^ in (**c**) simulation and (**d**) experiment.

For more detail regarding irradiance distribution between simulation and measurement, we compared two of them in the same coordinate in normalization in Fig. 6. The NCC was also calculated for each case, the height Z $=$ 25, 20, 15, 10, and 5 cm were 99.7, 99.2, 97.0, 95.1, and 95.6%, respectively, as shown in Fig. 6a–e. It is expressed that the simulation could effectively predict the optical performance in measurement. Furthermore, we simulated and measured more sections and then interpolated the values between obtained values. Finally, we achieved the irradiance distribution similar to the volume inside the box, as exhibited in Fig. 7a,b; the green color region represents the irradiance with a range from 6 to 9 W/m^2^ dominating in the box’s middle. Herein, we filtered the data with this range to observe its distribution inside the box, as shown in Fig. 7c,d. We emphasize that each element faces the top plate to receive the radiant flux from the top-down and the side plates of the box. In other words, if we locate an object inside the box with its surface upward, it could receive the light from the box top-down and the box side in the case without obstacle. Different object locations inside the box correspond to different irradiance that we figured out at various locations on the sections.

## UVC box treatments under in vitro conditions

The successful infection progress on host tissues of fungi C. musae consists of many steps such as rapid colonization, sporulation, conidial germination, and appressoria formation^36^. Hence, inactivating one of the steps among those could retard infection of *C. musae* and reduce disease development. In this study, we evaluated the effect of the UVC box on mycelial growth and conidial germination of species *C. musea*. Each experiment on the effects of UVC light in those cases was repeated three times.

Mycelial growth and conidial germination data were analyzed using Statistical Package for the Social Sciences software (SPSS Inc. 2000). Analysis of variance (ANOVA) was carried out to determine the significance of differences among means. The means of mycelial growth and conidial germination were compared using Fisher’s least significant difference (LSD). The inhibition rate of mycelial growth and conidial germination was calculated by using the formula.

3 $$I = \frac{{A_{0} - A_{1} }}{{A_{0} }} \times 100\% ,$$

where $I$ (%) is inhibition rate, ${A}_{0}$ is the diameter of the fungus colony (or the number of germinated conidia) in a medium without UVC radiation (control), ${A}_{1}$ is the diameter of the fungus colony (or the number of germinated conidia) in the presence of treatment^37^.

### Effect of UVC radiation on mycelial growth of species *C. musae*

In evaluating UVC box effects on mycelial growth, mycelial agar plugs (diameter of 5 mm) of *C. musae* were taken from 6 days old culture on PDA (Potato Dextrose Agar) plates and were transferred to clean PDA plates separately, which were opened and located in the middle of the UVC box with exposure time for 0 s, 30 s, 60 s, 2 min, 3 min, and 5 min, respectively. The UVC irradiance in middle section is around 6 W/m^2^, so this range of exposure time corresponds to UVC doses: 0.00 (control), 0.18, 0.36, 0.72, 1.08, and 1.80 kJ/m^2^. After treatments, the plates were incubated in a moisture chamber at 25 ℃. The colony with UVC treatment in each case was compared with the control sample by measuring the colony diameters after three days.

**Figure 8:**
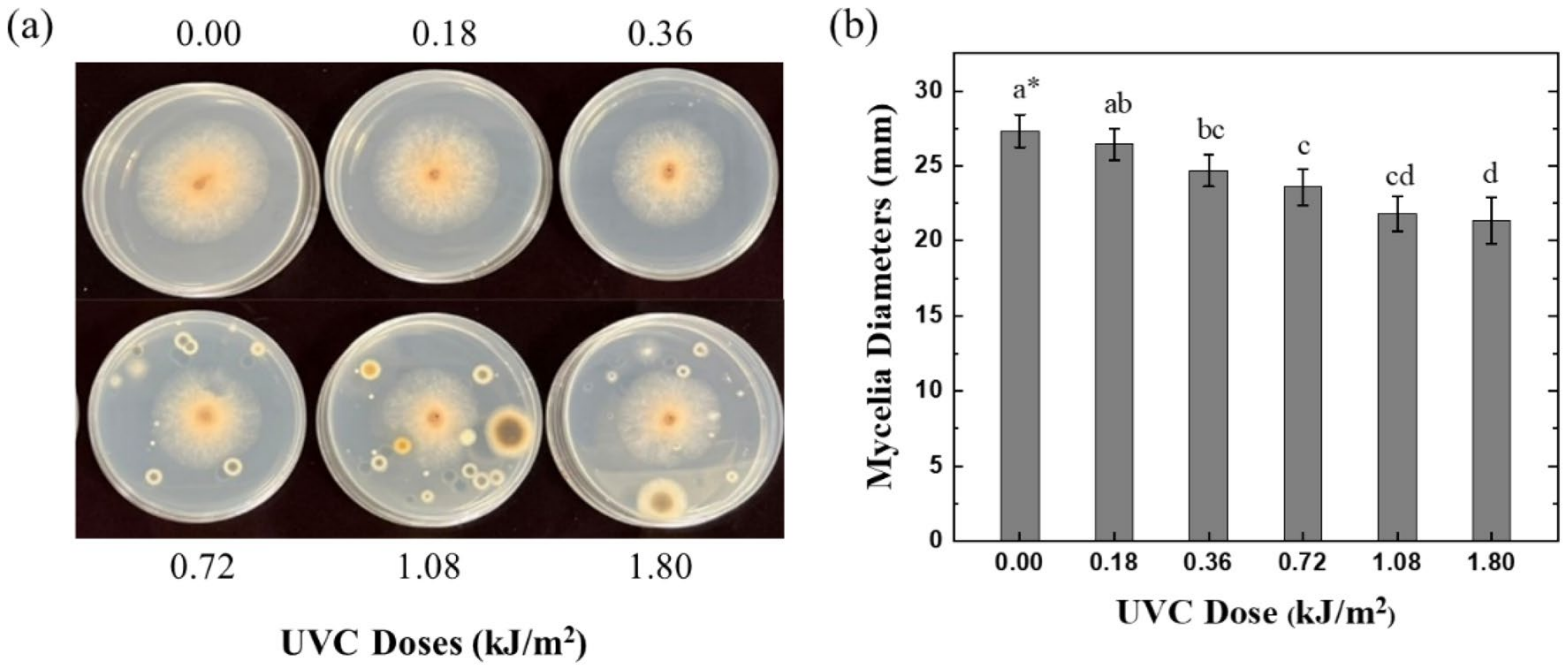
(**a**) The photos of fungi on PDA plates at different UVC doses and (**b**) the mycelial diameters of fungi at different UVC doses (* means within each column followed by the same letter are not significantly different at the 5.0% level according to LSD).

Overall, after three days of incubation, it was observed that the UVC radiation did not wholly inhibit the mycelial growth of *C. musae* within the range of evaluated doses. Figure 8a shows the photos of mycelial growth after three days, in which the colony diameters decreased gradually by increasing doses of UVC applied, as depicted in Fig. 8b. The inhibited effect was determined within the UVC dose range of 0.18–1.80 kJ/m^2^ compared to the control. However, the inhibition percentage of mycelial growth was less than 22% in selected UVC doses, as demonstrated in Table 1.

**Table 1:** Inhibition rate of fungi mycelial growth and conidial germination.

UVC dose (kJ/m^2^)	Mycelial growth (%)	Conidial germination (%)
0.18	3.2	69.3
0.36	9.7	91.2
0.72	13.7	92.0
1.08	20.2	99.3
1.80	21.9	98.5

### Effect of UVC radiation on conidial germination of species *C. musae*

In evaluating UVC box effects on conidial germination, conidia of *C. musae* were taken from 6 days old culture on PDA plates. Then they were made conidial suspensions (concentration of 10^6^ conidia/ml) separately and located in the middle of the UVC box with exposure time similar to the previous experiment for receiving different UVC doses: 0.00 (control), 0.18, 0.36, 0.72, 1.08, and 1.80 kJ/m^2^. After treatments, the suspensions were kept in a moisture chamber 25 ℃ for 24 h of incubation. Around 20 $\mathrm{\mu l}$ treated conidial suspension was dropped on the slides and examined under a microscope. The number of germinated conidia was recorded on a total of 100 conidia.

**Figure 9:**
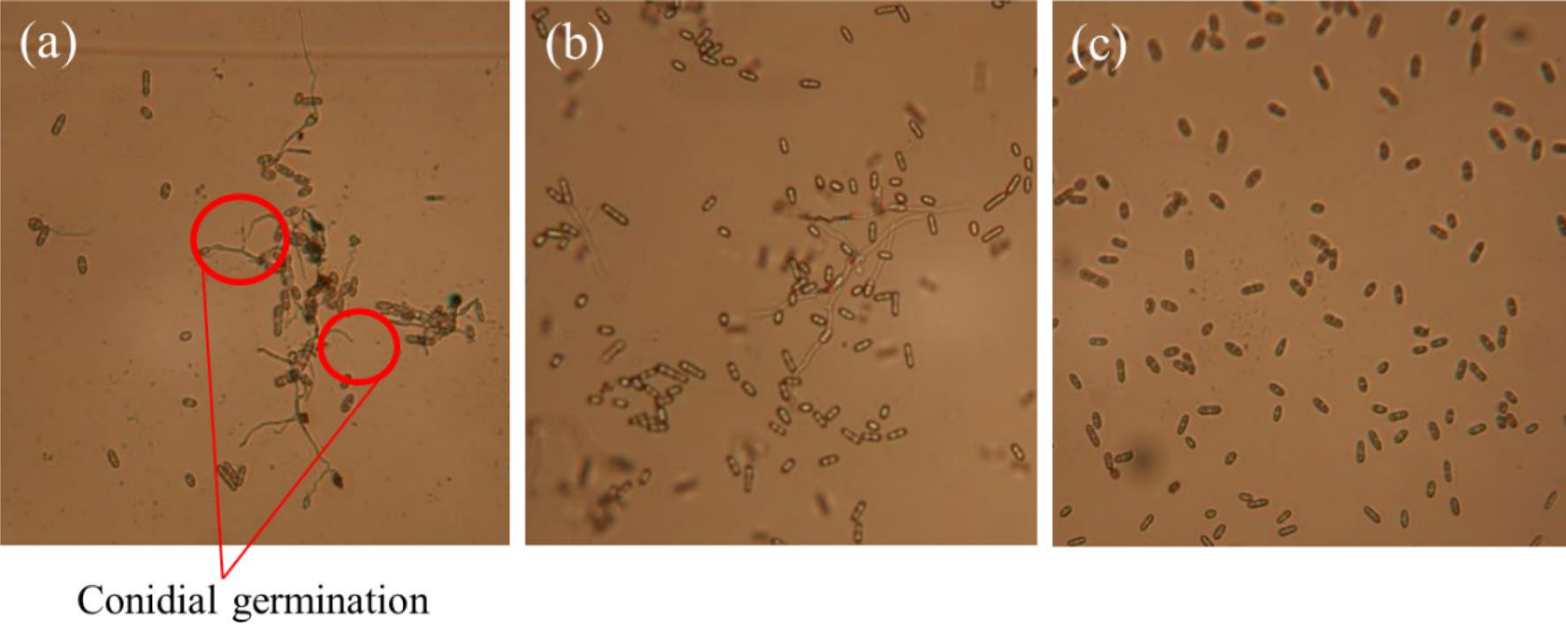
The photos of an amount of conidial suspension under the microscope at different UVC doses at (**a**) control, (**b**) 0.18, and (**c**) 1.08 kJ/m^2^.

**Figure 10:**
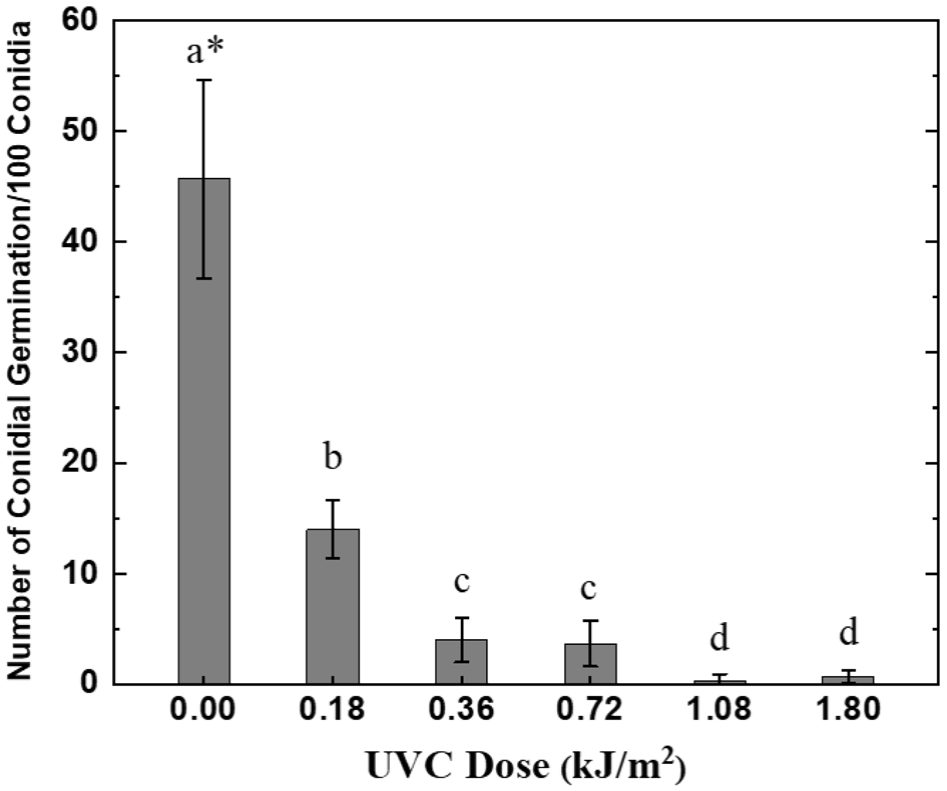
The statistical number of conidial germinations at different UVC doses (* means within each column followed by the same letter are not significantly different at the 5.0% level according to LSD).

After 24 h of incubation, germination of *C. musae* conidia was strongly inhibited by UVC radiation. Figure 9 shows the presentative photos of the UVC effect on conidial germination of *C. musae*. The conidial germination was observed clearly under the microscope for the control sample in Fig. 9a; they were sparser and shorter with 0.18 kJ/m^2^ UVC dose in Fig. 9b, and nearly unobserved with 1.08 kJ/m^2^ UVC dose in Fig. 9c. The results of conidial germinations in different selected UVC doses were summarized in Fig. 10, and the inhibition rates were calculated using Eq. (3), as shown in Table 1. Conidial germinations were partially inhibited with rates of 69.3% using UVC irradiation at the dose of 0.18 kJ/m^2^. They strongly inhibited over 90.0% at the dose of over 0.36 kJ/m^2^, with the highest inhibition rate up to 99.3% in applying the dose of 1.08 kJ/m^2^. However, in comparison of the UVC effect between the dose of 1.08 and 1.80 kJ/m^2^, the inhibition is not a big difference, which means the increase of the UVC dose might not inhibit 100.0% conidial germinations, as our prediction. According to the results, the UVC dose for *C. musae* conidia more than 0.36 kJ/m^2^ should have excellent inhibition effectiveness on conidial germination under vitro conditions.

## UVC treatment under in vivo conditions

**Figure 11:**
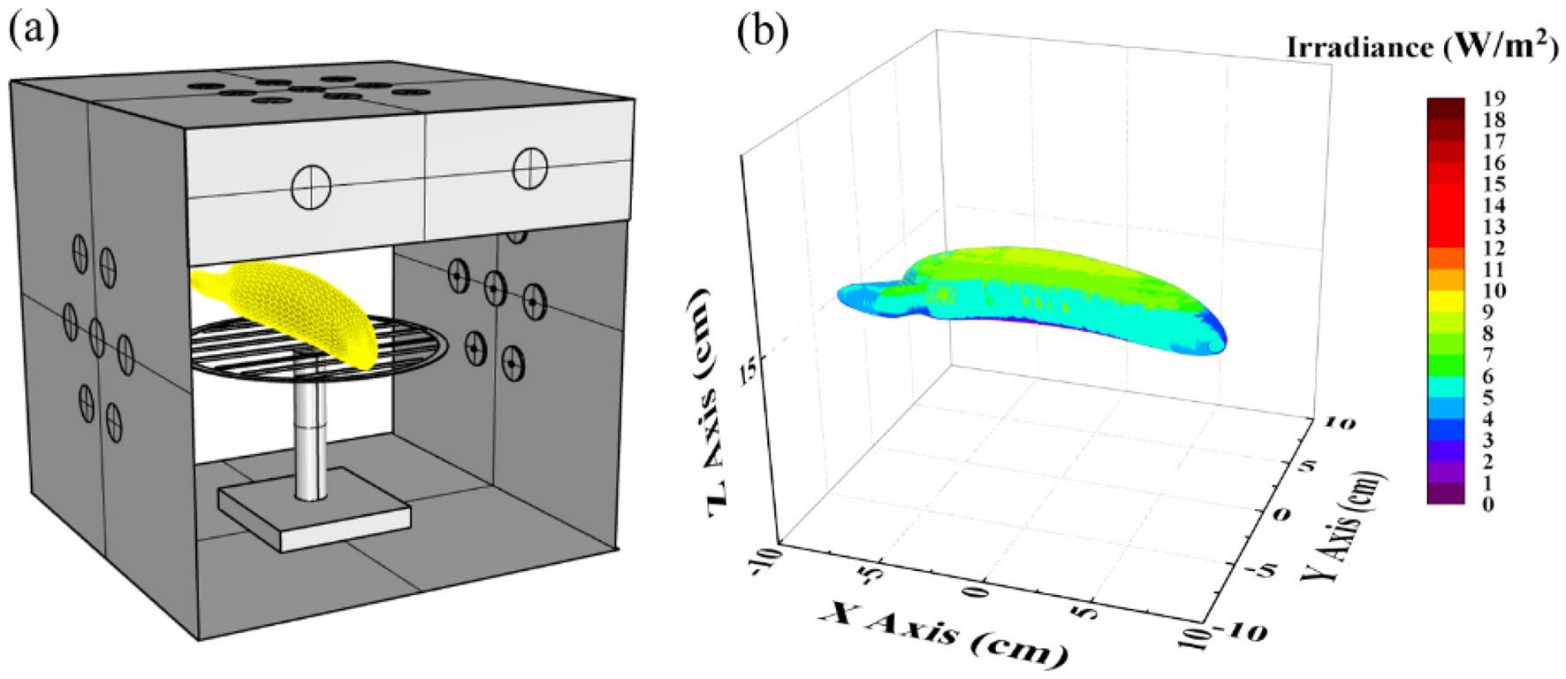
(**a**) Using a holder to locate a single banana in the middle of the UVC box, and (**b**) irradiance distribution on the banana object.

For more light to reach the banana peel, a single banana fruit was treated with UVC light by locating it in the box middle with the aid of a holder, as described in Fig. 11a. Although the irradiance distribution is displayed for various positions in Fig. 7, irradiance on a specific object could have different performances. It would be precious if we could approach the irradiation distribution on the surface of the banana. Nevertheless, there are still challenges in measuring the irradiance distribution on a particular banana fruit; meanwhile, the results of Sec. III show us that the simulated irradiance distribution is very similar to measurement, which leads to the inference that simulation could be valuable. In this case, we created a virtual banana model with dimensions nearly similar to banana fruit and then located it inside the designed box to predict the irradiance performance. The simulated irradiance results indicate that the top surface of the banana could receive an amount of UVC irradiance of around 6–9 W/m^2^, the side surface could receive an amount of about 5–6 W/m^2^, and the bottom surface could receive a small amount of UVC light, as shown in Fig. 11b.

**Figure 12:**
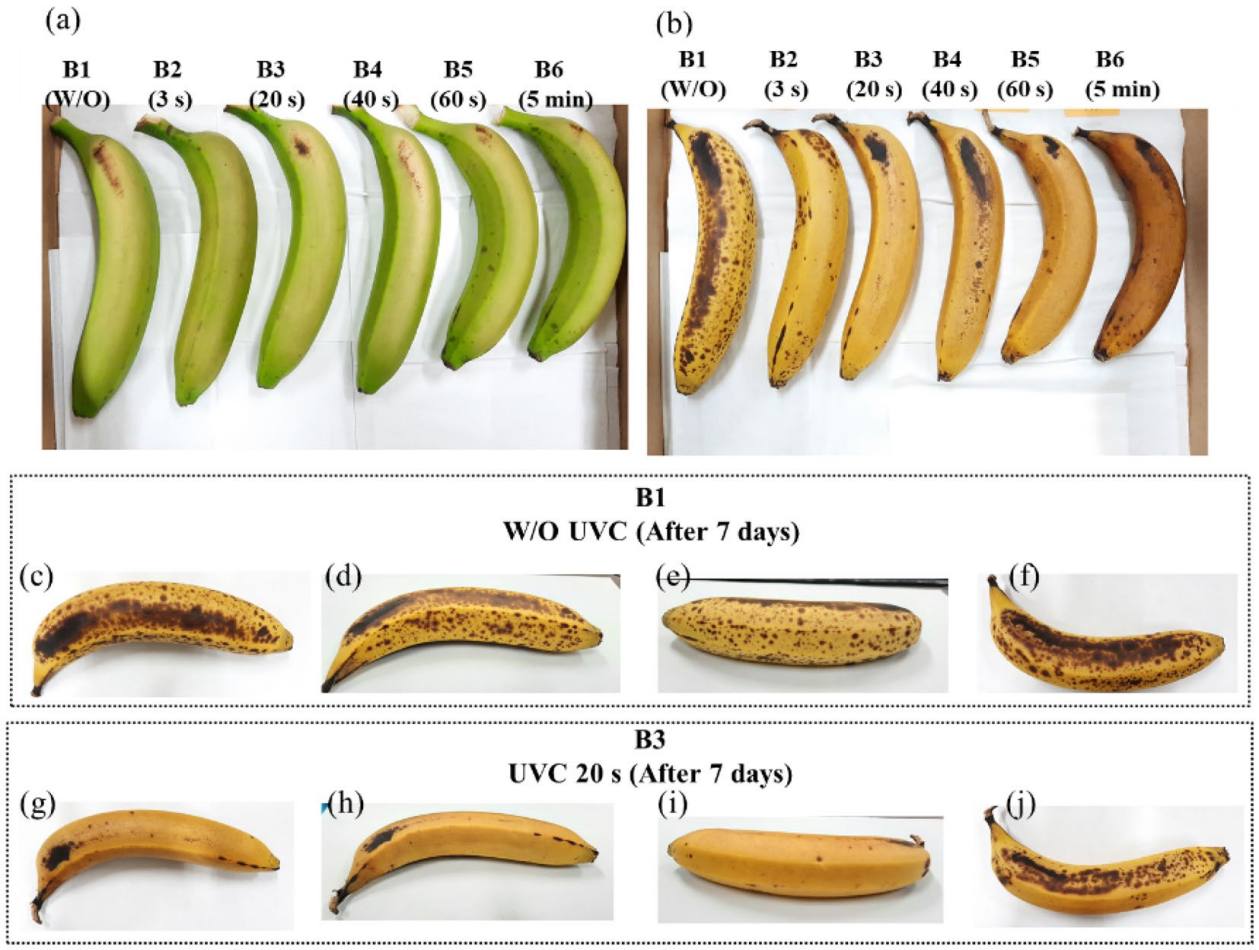
(**a**) Six banana samples were prepared for irradiation with UVC light at different exposure times, where samples B1, B2, B3, B4, B5, B6, B7 corresponded without UVC treatment (control), 3, 20, 40, 60 s, and 5 min, respectively; (**b**) after seven days from treatment time; sample B1 without treatment UVC light after seven days observed from (**c**) top view; (**d**,**e**) side view; and (**f**) bottom view; banana with treatment UVC light for 20 s after seven days observed from (**g**) top view; (**h**,**i**) side view; and (**j**) bottom view.

The bananas were selected from a local wholesale market in Tao-Yuan, Taiwan, so that the size and the shape were as uniform as possible and with minimal physical damage. They were transported to the laboratory on the same day, rinsed with tap water, and naturally dried at ambient temperature. Three trials were implemented with each UVC dose range to determine the optimal exposure time. Bananas for each trial were extracted from the same bunch of bananas. Initially, several bananas were prepared for treatment using the UVC box with different exposure times in Fig. 12a, where samples B1, B2, B3, B4, B5, and B6 were without UVC treatment (control), 3, 20, 40, 60 s, and 5 min, corresponding. All samples were stored at room temperature with a temperature of 23 °C, and the humidity around 85%. Figure 12b shows the results of the sensory properties of banana peel after seven days from treatment time; black spots of B2, B3, B4, B5, B6, and B7 were significantly reduced compared to the sample B1 via observation. However, over exposure time may cause the color of banana peel turns brown as B4, B5, and heavily on B6. This result shows that the suitable exposure time may range from 3 to 20 s for reducing black spots and minimal sensory damage to the banana peel. For more detail, the photos of all sides of sample B1 after seven days were taken, as shown in Fig. 12c–f; sample B3 in Fig. 12g–j. Observing sample B3 after seven days, although treated with the UVC box for 20 s, the banana bottom received the lowest UVC light, so the density of black spots still heavily appeared while was reducing on other sides, as shown in Fig. 12j, it is strongly demonstrated the effect of UVC light in reducing black spots for banana peel during storage.

**Figure 13:**
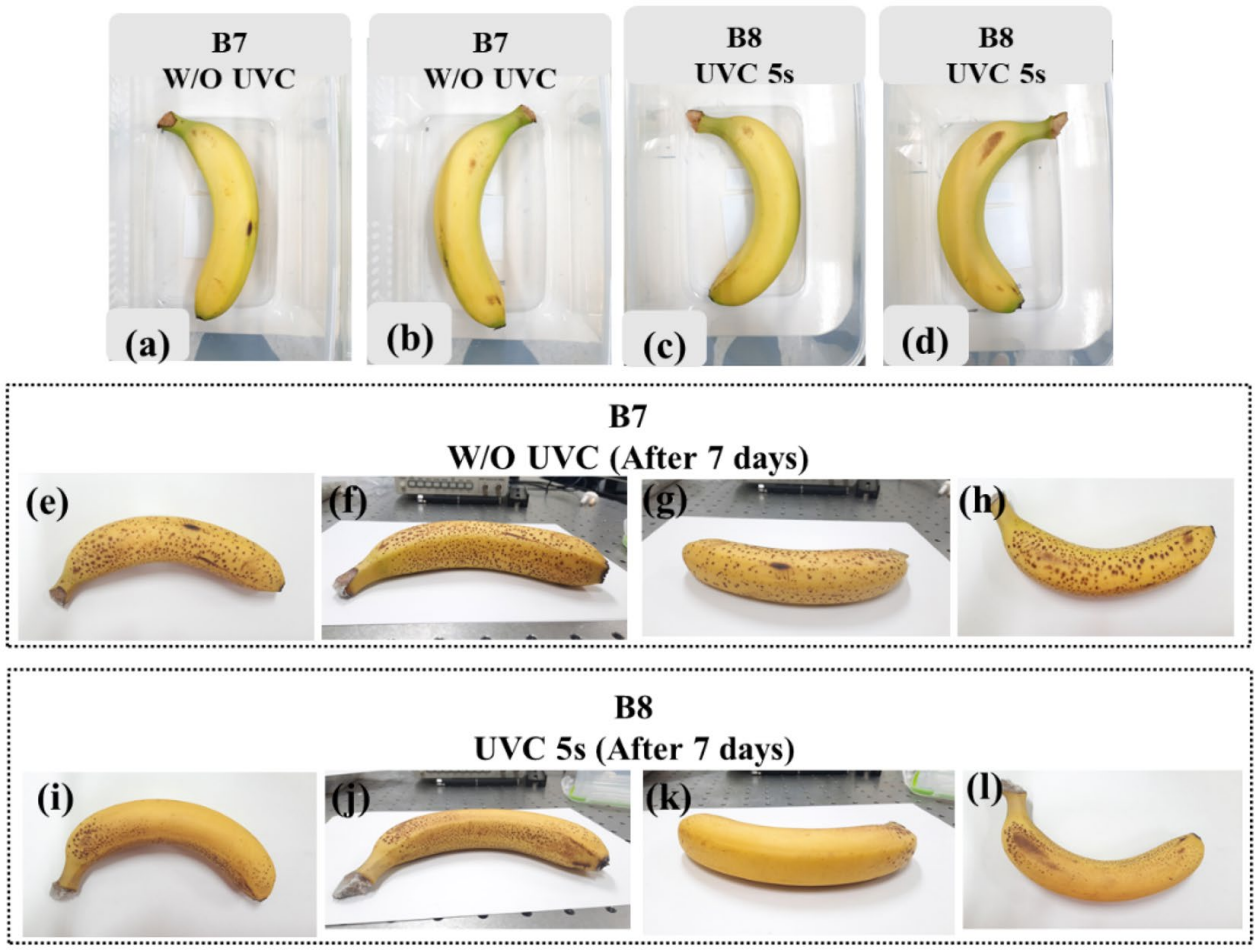
The photos of sample B7 without UVC treatment (**a**) top view, (**b**) bottom view; sample B8 was prepared for treatment with UVC light for 5 s observed from (**c**) top view, (**d**) bottom view; sample B7 after seven days observed (**e**) top view; (**f**,**g**) side view; and (**h**) bottom view; sample B8 after seven days observed from (**i**) top view; (**j**,**k**) side view; and (**l**) bottom view.

Many trials on banana fruits were respectively implemented to determine the suitable exposure time. In this work, the optimal exposure time for reducing black spots is about 5 s through observation. Figure 13a–d show banana samples B7 and B8 prepared for the treatment of control and 5 s, respectively. To enhance the UVC light given to the bottom side of the banana fruit, we faced up sample B8 and treated it a second time for 5 s. After seven days, the results revealed that the black spots were significantly reduced on sample B8 with limited sensory damage, as shown in Fig. 13e–l. According to the aforementioned result, the top surface of the banana model could receive an amount of UVC irradiance of around 6 to 9 W/m^2^. Besides, the suitable exposure time is 5 s to observe significantly-reduced black spots with minor damage to the banana surface. Finally, the optimum UVC dose is estimated from 0.030 to 0.045 kJ/m^2^ for the one-time upper treatment when considering the top surface of the banana. The higher UVC dose probably could harm banana peel. This finding was agreed by Mohamed et al.^27^, who observed that the treatment with the UVC dose higher than 0.050 kJ/m^2^ promotes rapid color change.

## Conclusions

Fabricating a system based on UVC LEDs is a potential option for reducing black spots on banana peels during storage that helps promote the economy of nations. Finding a suitable UVC dose, which is controlled by two critical parameters, irradiance and exposure time, is crucial. The UVC dose should be enough to delay black spots on a banana peel. However, banana peels are very sensitive to UVC because overdose will cause damage to banana skin. Therefore, monitoring the irradiance distribution and finding the suitable UVC dose is essential when fabricating a specific sterilization product.

In this research, by applying the precise mid-field light source model that we achieved, the 275 nm UVC LED box with dimensions of 30 $\times$ 30 $\times$ 30 cm was designed with high irradiance uniformity on the middle section of the box. The box was fabricated by arranging UVC LED similar to simulation on the box coved by Aluminum. The irradiance was carefully monitored in both simulation and measurement on many sections that receive light from the box top downward and sides. The result indicated that simulation could effectively predict the optical behavior because irradiance distribution in the simulation is virtually similar to measurement. The dominant irradiance is distributed in the middle region of the box with a range of 6 to 9 W/m^2^.

Under in vitro conditions, the effectiveness of the UVC box was proved in the inhibition species fungal *C. musae*, in which mycelial growth at the UVC dose of 1.80 kJ/m^2^ is inhibited at 21.9% in comparison with control after three days, while with 0.36 kJ/m^2^ or above of UVC dose could be got excellent inhibition effectiveness as over 90% on conidial germination after 24 h. Finding inhibition effects on mycelial growth and conidial germination would decrease infection of fungal *C. musae* and disease development. In vivo treatment, to find the suitable UVC dose, we conducted many trials on banana fruits by locating a single banana in the middle of the fabricated box. In this way, we created a banana model and located it inside the designed box to predict irradiance distribution on the banana peel. The upper surface irradiance distribution ranged from 6 to 9 W/m^2^. The results indicated that an exposure time of about 5 s is best for reducing black spots with minimal damage to peel tone through observation. Finally, the low UVC dose is proposed from 0.030 to 0.045 kJ/m^2^ for the one-time upper treatment when considering the top surface of the banana. This finding also supports fabricating a sterilization system with other dimensions suitable for different users. Consequently, this research offers a potential application for factory conveyor belts or households with high effectiveness, convenience, and flexibility in reducing the seriousness of black spots in postharvest bananas.

## Data Availability

All datasets from this study are available from the corresponding author upon reasonable request.

## References

[1] World Health Organization, *Ultraviolet Radiation* (Environmental Health Criteria 160, 1994).

[2] Sastry SK, Datta AK, Worobo RW (2000). Ultraviolet light. J. Food Sci..

[3] Giese, A. C. Ultraviolet radiation. In *Encyclopedia of Physical Science and Technology* 19–20 (McGraw-Hill, 1992).

[4] Costa L, Vicente AR, Civello PM, Chaves AR, Martinez GA (2006). UV-C treatment delays postharvest senescence in broccoli florets. Postharvest Biol. Technol..

[5] Stevens C (1998). Application of hormetic UV-C for delayed ripening and reduction of rhizopus soft rot in tomatoes: The effect of tomatine on storage rot development. J. Phytopathol..

[6] De Capdeville G, Wilson CL, Beer SV, Aist JR (2002). Alternative disease control agents induce resistance to blue mold in harvested ‘Red Delicious’ apple fruit. J. Phytopathol..

[7] Spadoni, A., Neri, F. & Mari, M. Physical and chemical control of postharvest diseases. In *Advances in Postharvest Fruit and Vegetable Technology* 89–116 (CRC Press, 2015).

[8] Eckert JW, Ogawa JM (1985). The chemical control of postharvest diseases: Subtropical and tropical fruits. Annu. Rev. Phytopathol..

[9] Wilson CL, Wisniewski ME (1989). Biological control of posthravest diseases of fruits and vegetables: An emerging technology. Annu. Rev. Phytopathol..

[10] Gullino ML, Kuijpers LAM (1994). Social and political implications of managing plant diseases with restricted fungicides in Europe. Annu. Rev. Phytopathol..

[11] Ragsdale NN, Sisler HD (1994). Social and political implications of managing plant diseases with decreased availability of fungicides in the United States. Annu. Rev. Phytopathol..

[12] Mari M, Guizzardi M (1998). The postharvest phase: emerging technologies for the control of fungal diseases. Phytoparasitica.

[13] Janisiewicz WJ, Korsten L (2002). Biological control of postharvest diseases of fruits. Annu. Rev. Phytopathol..

[14] Terry LA, Joyce DC (2004). Elicitors of induced disease resistance in postharvest horticultural crops: A brief review. Postharvest Biol. Technol..

[15] Turtoi M (2013). Ultraviolet light treatment of fresh fruits and vegetables surface: A review. J. Agroaliment. Process. Technol..

[16] Gayas, B., Munaza, B. & Sidhu, G. K. Ultraviolet light treatment of fresh fruits and vegetables. In *Processing of Fruits and Vegetables: From Farm to Fork* 83–100 (CRC Press, 2019).

[17] Pombo MA, Rosli HG, Martinez GA, Civello PM (2010). UV-C treatment affects the expression and activity of defense genes in strawberry fruit (*Fragaria ananassa*, Duch.). Postharvest Biol. Technol..

[18] Gonzalez-Aguilar GA, Villa-Rodriguez JA, Ayala-Zavala JF, Yahia EM (2010). Improvement of the antioxidant status of tropical fruits as a secondary response to some postharvest treatments. Trends Food Sci. Technol..

[19] Zukauskas A, Shur MS, Caska R (2002). Introduction to Solid-state Lighting.

[20] Steigerwald DA (2002). Illumination with solid state lighting technology. IEEE J. Sel. Top. Quant..

[21] Robinson JC (1996). Crop Production Science in Horticulture (5): Bananas & Plantains CAB International.

[22] Basel, R. M., Racicot, K. & Senecal, A. G. Long shelf life banana storage using MAP storage coupled with postharvest MCP treatment. In *Annual Meeting and Food Expo-Anaheim*, California, USA, 15–19 (2002).

[23] Wardlaw CW (1961). Banana Diseases Including Plantains and Abaca.

[24] Jeffries P, Dodd JC, Jeger MJ, Plumbley RA (1990). The biology and control of *Colletotrichum* species on tropical fruit crops. Plant Pathol..

[25] Pongprasert N, Sekozawa Y, Sugaya S, Gemma H (2011). A novel postharvest UV-C treatment to reduce chilling injury (membrane damage, browning and chlorophyll degradation) in banana peel. Sci. Hortic..

[26] Mohamed NT, Ding P, Ghazali HM, Kadir J (2017). Biochemical and cell wall ultrastructural changes in crown tissue of banana (Musa AAA ‘Berangan’) fruit as mediated by UVC irradiation against crown rot fungal infection. Postharvest Biol. Technol..

[27] Mohamed NT, Ding P, Kadir J, Ghazali HM (2017). Potential of UVC germicidal irradiation in suppressing crown rot disease, retaining postharvest quality and antioxidant capacity of Musa AAA “Berangan” during fruit ripening. Food Sci. Nutr..

[28] Sun CC, Lee TX, Ma SH, Lee YL, Huang SM (2006). Precise optical modeling for LED lighting verified by cross correlation in the midfield region. Opt. Lett..

[29] Sun CC, Chien WT, Moreno I, Hsieh CC, Lo YC (2009). Analysis of the far-field region of LEDs. Opt. Express.

[30] Le TTN (2021). Precise mid-field modeling for UVC LEDs by using a fluorescent film. OSA Contin..

[31] Sun CC, Lee TX (2021). Optical Design for LED Solid State Lighting: A Guide.

[32] ASAP Program. *Breault Research Organization (BRO), Inc.* http://www.bro.com/S.

[33] Shih BJ (2015). Study of temperature distributions in pc-WLEDs with different phosphor packages. Opt. Express.

[34] Yang TH (2018). Noncontact and instant detection of phosphor temperature in phosphor-converted white LEDs. Sci. Rep..

[35] Lee HW, Lin BS (2012). Improvement of illumination uniformity for LED flat panel light by using micro-secondary lens array. Opt. Express..

[36] De Costa DM, Gunawardhana HMDM (2012). Effects of sodium bicarbonate on pathogenicity of *Colletotrichum musae* and potential for controlling postharvest diseases of banana. Postharvest Biol. Technol..

[37] Harlapur SI, Kulkarni MS, Wali MC, Srikantkulkarni H (2010). Evaluation of plant extracts, bio-agents and fungicides against *Exserohilum turcicum* (Pass.) Leonard and Suggs. causing Turcicum leaf blight of maize. Karnataka J. Agric. Sci..

